# Role of Multidisciplinary Team Meetings in the Diagnosis and Management of Diffuse Parenchymal Lung Diseases in a Tertiary Care Hospital

**DOI:** 10.1055/s-0043-1776063

**Published:** 2023-11-01

**Authors:** Mohammad Ayaz Khan, Nahid Sherbini, Sami Alyami, Abdullah Al-Harbi, Suliman Alrajhi, Reem Abdullah, Dhafer AlGhamdi, Rajkumar Rajendram, Hana Bamefleh, Hamdan Al-Jahdali

**Affiliations:** 1 College of Medicine, King Saud Bin Abdulaziz University for Health Sciences, Riyadh, Saudi Arabia; 2 King Abdullah International Medical Research Centre, Riyadh, Saudi Arabia; 3 Division of Pulmonary, Ministry of National Guard-Health Affairs, Department of Medicine, Riyadh, Saudi Arabia; 4 Internal Medicine Division, Ministry of National Guard-Health Affairs, Department of Medicine, Madinah, Saudi Arabia; 5 Department of Imaging, Ministry of National Guard-Health Affairs, Riyadh, Saudi Arabia; 6 Internal Medicine Division, Ministry of National Guard-Health Affairs, Department of Medicine, Riyadh, Saudi Arabia; 7 Department of Pathology and laboratory, Ministry of National Guard-Health Affairs, Riyadh, Saudi Arabia

**Keywords:** multidisciplinary team, diffused parenchymal lung diseases, interstitial lung disease, idiopathic pulmonary fibrosis, usual interstitial pneumonia, chronic hypersensitivity pneumonitis

## Abstract

**Background** Decisions on the management of interstitial lung diseases (ILD) and prognostication require an accurate diagnosis. It has been proposed that multidisciplinary team (MDT) meetings for ILD (ILD-MDT) improve these decisions in challenging cases of ILD. However, most studies in this field have been based on the decisions of individual clinicians and there are few reports on the outcomes of the ILD-MDT approach. We therefore describe the experience of the ILD-MDT meetings at our institution.

**Methods** A single-center retrospective review of the electronic health care records of patients discussed in the ILD-MDT meetings at our institution from February 2016 to January 2021 was performed. At out institution, at each ILD-MDT meeting, the referring pulmonologist presents the clinical history and the results of all relevant investigations including serology, blood gas analyses, lung function tests, bronchoscopy, and bronchoalveolar lavage. A radiologist then describes the imaging including serial computed tomography (CT) scans. When available, the findings on lung biopsy are presented by a pathologist. Subsequent discussions lead to a consensus on the diagnosis and further management.

**Results** The study included 121 patients, comprising 71 (57%) males and 76 nonsmokers (62.8%), with a mean age of 65 years (range: 25–93 years). The average number of comorbidities was 2.4 (range: 0–7). Imaging-based diagnoses were usual interstitial pneumonia (UIP)/chronic hypersensitivity pneumonitis (CHP) in 32 (26%) patients, UIP in 20 (17%) patients, probable UIP in 27 (22%) patients, nonspecific interstitial pneumonia in 11 (9%) patients, and indeterminate interstitial lung abnormalities (ILA) in 10 (8%) patients. The most common consensus clinical diagnosis after an ILD-MDT discussion was chronic hypersensitivity pneumonitis/idiopathic pulmonary fibrosis in 17 patients (14%), followed by idiopathic pulmonary fibrosis and connective tissue disease associated interstitial lung disease in 16 patients (13%), CHP in 11 patients (9.1%), and ILA in 10 patients (8.4%). Only a 42 patients (35%) required surgical lung biopsy for confirmation of the diagnosis.

**Conclusion** This study describes the characteristics of the patients discussed in the ILD-MDT meetings with emphasis on their clinical, radiological, and laboratory data to reach a diagnosis and management plan. The decisions on commencement of antifibrotics or immunosuppressive therapy for patients with various ILDs are also made during these ILD-MDT meetings. This descriptive study could help other health care professionals regarding the structure of their ILD-MDT meetings and with discussions about diagnostic and care decisions for diffused parenchymal lung disease patients.

## Introduction and Background

Diffused parenchymal lung diseases (DPLDs), otherwise known as interstitial lung diseases (ILD), are a diverse group of respiratory conditions characterized by damage, fibrosis, and inflammation of the lungs. 1 The most common and well studied of these is idiopathic pulmonary fibrosis (IPF). IPF is a devastating, progressive disease that requires careful and early identification. Further study and characterization of all ILDs is needed to reach better agreement on diagnosis and treatment decisions. In 2018, the American Thoracic Society/European Respiratory Society (ATS/ERS) guidelines suggested the discussion of patients' clinical data by a team of specialists in ILD multidisciplinary team (ILD-MDT) meetings to increase diagnostic certainty. 2 These ILD-MDT meetings can increase the accuracy of diagnoses or at least help formulate a working diagnosis.

In one study, ILD-MDT meetings modified the diagnoses of around 50% of patients and these diagnoses were consistent with the outcomes of each patient. 3 Another global study of ILD patients reflected how these MDT meetings increased diagnostic agreement and reproducibility. These meetings even brought the prognostic performance of nonuniversity academics to the level of IPF experts. 4

Few data are available on the processes and outcomes of ILD-MDT meetings in Saudi Arabia. One study describing ILD in Saudi Arabia reported connective tissue disease (CTD) related ILD to be the most common ILD. 5 Another study describing ILD in Saudi Arabia concluded that IPF affects older adults and progressed slower in their cohort than in other populations. 6 However, this study focused on patients' pathological diagnoses 6 rather than consensus diagnoses. In the 21st century, the management of ILD remains challenging, and clinicians, teams, and institutions vary in their approaches to ILD. We therefore present the data from our ILD-MDT meetings to inform regional and international clinicians about the approach to ILD at King Abdulaziz Medical City, Riyadh (KAMC).

## Subjects and Methods

## Materials and Methods

KAMC is a large regional academic tertiary care center with dedicated ILD clinics run by a team of pulmonologists with pulmonary rehabilitation services. The ILD service at KAMC was established in 2016 with subspecialty clinics, support from allied health care professionals, and ILD-MDT meetings. The ILD-MDT panel includes a team of five pulmonologists, a chest pathologist, a rheumatologist, and a chest radiologist, all of whom have subspecialty interests in DPLD. We report our experience of these ILD-MDT meetings to describe the diagnostic and management decisions reached by consensus in these meetings.

Rheumatologists and thoracic surgeons attend meetings if and when required. All challenging cases are discussed in this forum to achieve a consensus on diagnosis, treatment decisions, and follow-up plans.

The referring clinicians would present the clinical history and relevant investigations including the results of blood tests, serology, lung function, 6-minute walk tests (6MWT), echocardiography, bronchoscopy, and bronchoalveolar lavage (BAL) data. The radiologist would present the radiological data including serial computed tomography (CT) scans. Discussions led to a consensus diagnosis or requests for additional investigations such as lungs biopsies. Cases with a histopathological diagnosis were rediscussed and a working diagnosis would be formulated. The treatment decision would then be agreed upon and documented in the patient's electronic health care record. The decisions were conveyed to patients and relatives in clinics and following their agreement the treatment would commence. The patients were then followed closely to monitor their response to treatment and side effects as well as review decisions that needed to be changed for future treatments. The follow-up visits and subsequent progress on management decisions, although desirable, are beyond the scope of this study due mainly to logistics, preference of individual clinician in rediscussing their cases, and lack of sufficient data for such reporting. We, however, are working on a registry for all our patients with DPLDs and will report on the outcomes of all initial and subsequent progress or change of diagnoses in due course. Here the nature of the descriptive study is to report our initiation and progress of ILD-MDT meetings as the prototype in the kingdom and what the structures of these meetings were.

This is a single-center retrospective review of patients' electronic health care records and MDT reports over a 5-year period from February 2016 to January 2021. Only adult patients (≥18 years) whose cases were discussed in the ILD-MDT meetings were included. Experienced clinicians extracted data from the patient's electronic record after approval from the institutional research board (IRB). Those without complete datasets were excluded. All the data were held in a centralized, encrypted database.

### Statistical Analyses

Statistical analyses of demographics, laboratories, lung function, investigative data, treatments, clinical outcomes, and mortality data have been tabulated. The mean, standard deviation, and range were used to describe the patient demographics and the radiological and physiological data. Frequencies and percentages were used to describe the categorical data.

## Results

The analysis included 121 patients (mean age: 65 years; age range: 25–93 years; male: 71, 57%; nonsmokers: 76, 63%). The average body mass index (BMI) was 29 ± 12 kg/m ^2^ . The average number of comorbidities was 2.4 (range: 0–7). The most common comorbidities were diabetes mellitus (DM), followed by hypertension (HTN), dyslipidemia (DLP), and ischemic heart disease (IHD).

Lung function data were available for 72 patients (60%; Table 1 ). The remaining data were either incomplete or the lung function tests could not be performed in the patients. The mean forced expiratory volume over 1 second (FEV1) was 60%, mean forced vital capacity (FVC) was 65%, and the mean total lung capacity (TLC) was 60%. Eighty patients (66%) were able to complete the 6MWT. Desaturation (<90%) occurred in 53 patients (44%). The echocardiographic data in this cohort found that 65 patients (55%) had preserved left ventricular function. Left ventricular systolic dysfunction with reduced ejection fraction was found in 46 patients (38%). The ejection fraction was less than 40% in 10 patients (8%). The right ventricular systolic pressure was within the reference range in 76 (63%) patients. The right ventricular systolic pressure was raised in 45 patients (37%), categorized as 30 to 40 mm Hg in 23 patients (51%), 40 to 50 mm Hg in 13 patients (29%), and 50 to 60 mm Hg in 9 patients (20%).

**Table 1:** Clinical characteristics of the cohort

Total number of patients, *N* = 121	
Characteristic	Number (%)
Male	71 (57)
Nonsmokers	76 (63)
**FEVI**	
Able to do	72 (60)
Mean (range)	60% (37–106)
**FVC**	
Able to do	72 (60)
Mean (range)	65% (4–98)
**TLC**	
Able to do	66 (55)
Mean (range)	60% (40–87)
** 6MWT O _2_ desaturation <90% **	
Able to do	53 (44)
Unable	68 (56)
**ABG**	
Available	37 (31)
Unavailable	84 (69)
**Echo RV pressure (mmHg)**	
Normal	76 (63)
30–40	23 (19)
40–50	13 (11)
50–60	9 (7)

Abbreviations: 6MWT, 6-minute walk test; ABG, arterial blood gas; FEVI, forced expiratory volume in the first second; FVC, forced vital capacity; RV pressure, right ventricular pressure; TLC, total lung capacity.

The radiological diagnoses based on the chest CT scan findings are listed in Table 2 . These were the following: usual interstitial pneumonia (UIP)/chronic hypersensitivity pneumonitis (CHP) in 32 patients (26%), UIP in 20 patients (17%), probable UIP in 27 patients (22%) and nonspecific interstitial pneumonia (NSIP) in 11 patients (9%). The radiological appearance was indeterminate in 10 patients (8%). Only 42 patients (35%) had a surgical lung biopsy to determine the diagnosis. Serology was negative for all relevant autoantibodies in 56 patients (46%). The most commonly identified autoantibodies were antismooth muscle antibodies (ASMA) in 27 patients (22%), antinuclear antibodies (ANA) in 15 patients (13%) and rheumatoid factor (RF) in 10 patients (8%).

**Table 2:** Demographic data of the cohort

Total number of patients, *N* = 121	
Characteristic	Number (%)
Mean age: 65 y (range: 25–93 y)	
Mean BMI (± SD): 29 ± 12	
**Radiologist HRCT diagnosis**	
UIP/CHP	32 (26%)
UIP	20 (17%)
Probable UIP	27 (22%)
NSIP	11 (9%)
Indeterminate	10 (8%)
Organizing pneumonia	7 (6%)
CPFE	7 (6%)
Alternative/CHP	7 (6%)
**Surgical lung biopsy**	
Done	42 (35%)
Not done	79 (65%)
**Serology**	
Negative	56 (46%)
ASMA	27 (22%)
ANA	15 (13%)
RF positive	10 (8%)
Anti-CCP IgG	2 (1.7%)
Low c3c4 high RF	2 (1.7%)
Miscellaneous (Anti-SCL70)	3 (2.5%)

Abbreviations: Anti-CCP, anticyclic citrullinated peptide antibody; ASMA, antismooth muscle antibodies; ANA, antinuclear antibodies; BMI, body mass index; CHP, chronic hypersensitivity pneumonitis; CPFE, combined pulmonary fibrosis with emphysema; HRCT, high-resolution CT scan; IgG, immunoglobulin G, NSIP, nonspecific interstitial pneumonitis; RF, rheumatoid factor; SD, standard deviation; UIP, usual interstitial pneumonia.

The clinical diagnoses made after the ILD-MDT discussion are listed in Table 3 . The commonest were CHP/IPF in 17 patients (14%), IPF in 16 patients (13%), CTD-ILD in 16 patients (13%), and CHP in 11 patients (9.1%). Indeterminate and interstitial lung abnormalities (ILA) were reported in approximately 8.4% of patients.

**Table 3:** Consensus diagnosis in ILD-MDT meetings

Diagnosis	No. of patients, *n* (%)
CHP/IPF	17 (14)
CTD-ILD	16 (13.5)
IPF	15 (12.5)
CHP	11 (9.1)
Indeterminate	10 (8.4)
ILA	10 (8.4)
IPF/CPFE phenotype/familial type	7 (6.0)
Idiopathic NSIP	6 (5)
Smoking-related ILD	5 (4.1)
Chronic organizing pneumonia	5 (4.1)
Probable CHP	4 (3.3)
Chronic eosinophilic pneumonia	4 (2.5)
Organizing pneumonia	2 (1.7)
Sarcoidosis	2 (1.7)
Acute HP	1 (0.8)
Alveolar hemorrhage	1 (0.8)
Drug induced: MTX ILD	1 (0.8)
Drug induced: tacrolimus pneumonitis	1 (0.8)
Drug induced: methotrexate pneumonitis	1 (0.8)
Lipoid pneumonia with PAH	1 (0.8)
PPFE	1 (0.8)

Abbreviations: CHP/IPF, chronic hypersensitivity pneumonitis/idiopathic pulmonary fibrosis; CPFE, combined pulmonary fibrosis with emphysema; CTD-ILD, connective tissue disease related ILD; HP, hypersensitivity pneumonitis; ILD, interstitial lung disease; MDT, multidisciplinary team; MTX-ILD, methotrexate induced ILD; NSIP, nonspecific interstitial pneumonitis; PAH, pulmonary alveolar hemorrhage; PPFE, pleuroparenchymal fibroelastosis.

The treatment decisions made after the ILD-MDT discussions are summarized in Table 4 . The most commonly recommended treatment approach based on the information presented at the MDT meetings was a combination of anti-inflammatory treatment with steroids and immunosuppression (i.e., steroids/immunosuppression; 42 patients, 35%). In about a fifth of the cohort, the available data precluded a clear diagnosis from being made. For practical purposes, the ILD-MDT recommended that these patients be started on anti-inflammatory treatment and followed closely to observe the linear behavior of the disease. The ILD-MDT team also recommended that these patients be offered antifibrotic therapy in the event of progression. The decision to add antifibrotic medication would be at the discretion of the treating physician. It did not require further discussion. So these data were not recorded in the ILD-MDT meetings. Thus, it is likely that many patients will have subsequently switched to antifibrotic therapy.

**Table 4:** Management decisions based on diagnosis of the cohort

Total number of patients, *N* = 121	
MDT treatment decision	
Steroids/immunosuppression	42 (35%)
Steroids/immunosuppression followed by antifibrotic if there is progress	24 (20%)
Antifibrotic drugs	22 (18%)
Wait and watch	19 (15%)
Steroids, short course	14 (12%)

Antifibrotic treatment for IPF (the only indication at the time) was prescribed for 22 patients (18%). A “wait and watch” policy was adopted in 19 patients (15%) with stable lung function, 6MWT, and post-MDT discussion with patients and their relatives. The most common pharmacological combination therapies were prednisolone with mycophenolate mofetil (Pred-MMF) in 42 patients (35%), antifibrotic agents in 22 patients (18%), and prednisolone with azathioprine (Pred-azathio) in 18 patients (15%). Proton pump inhibitors were used in 106 patients (88%) for symptoms of gastroesophageal reflux disease (GERD; Table 5 ). Patients who were hypoxic at rest or on exertion (i.e., O _2_ saturation < 88%) were provided with oxygen therapy (33 patients, 27%) and/or pulmonary rehabilitation (43 patients, 36%).

**Table 5:** Pharmacological agents used in cohort

Total number of patients, *N* = 121	
Characteristic	No. of patients (percentage)
Drug name	
Prednisolone-mycophenolate mofetil (MMF)	42 (35)
Antifibrotic drugs	22 (18)
Prednisolone-azathioprine	18 (15)
Wait and watch policy	19 (15)
Prednisolone only	14 (12)
Prednisolone/cyclophosphamide/MMF	1 (0.8)
Steroids and cyclophosphamide	5 (4)
**PPI use**	
Yes	106 (88)
No	15 (12)
**Oxygen therapy**	
Yes	33 (27)
No	88 (73)

Abbreviation: PPI, proton pump inhibitor.

## Discussion

This article provides a comprehensive overview of the role of MDT meetings in the diagnosis and treatment of DPLD at our institution. These meetings had a significant impact on the management of this cohort. Our data reinforce the importance of an accurate and timely diagnosis and the benefits of a multidisciplinary approach. We also highlight the challenges of coordinating care across several specialties.

The optimal setup of an ILD-MDT had not been described when these meetings were started at our institution. Thus, the initial format was modified from that of the lung cancer tumor board meetings. There was a learning curve for the conduct of the ILD-MDT meetings at our institution until the currently used pathway became established after approximately 12 meetings over 6 months.

Previous studies on ILD were based on the decisions of individual clinicians rather than a multidisciplinary consensus. 7 8 9 Thus, the initial guidelines on the management of ILD recommended the performance of a surgical lung biopsy for patients with a clinical diagnosis of possible or probable UIP, and those with atypical features. 10 However, a surgical lung biopsy carries significant risk in this patient population due to the severity of their disease and their comorbidities. 11 12 13

Recent guidelines have moved away from the use of surgical lung biopsy. The use of clinical and radiological data in conjunction with bronchoalveolar lavage to reach a consensus diagnosis in MDT meetings is considered preferable. 14 15 In the present cohort, a surgical lung biopsy was only performed when the ILD-MDT could not reach a consensus about the diagnosis (45 patients, 35%).

The most common ILD-MDT consensus diagnoses in the present cohort were CHP overlapping with IPF (17 patients, 14%) and CTD-ILD (16 cases, 13.5%). The age range of the cohort (25–90 years) also reflects the various possible diagnoses reported in our cohort, such as organizing pneumonia (OP), smoking-related ILD, and NSIP. These observations are consistent with previously reported epidemiological studies. 16 17 In our cohort, 8% of ILD cases were indeterminate or unclassifiable, closely mirroring Ryerson et al's study 18 in which 10% were indeterminate. The specific pathological diagnosis is in almost 23% of the cases, such as chronic eosinophilic pneumonia, drug-induced ILD, OP, and NSIP.

A previous Saudi study reported the prevalence of various ILDs. 5 The most common causes of DPLD in that cohort were CTD-associated ILD (34.8%), IPF (23.3%), sarcoidosis (20%), and hypersensitivity pneumonitis (HP; 6.3%). 5 Another study 19 found that CTD-ILD was the most common cause of DPLD. This was the second most common cause in the present cohort. The final ILD diagnoses in the present cohort differ from the global prevalence of ILD. This may be due to discordance in clinical data, radiological appearances, or a lack of histopathological data.

In the present study, 33 patients (27.27%) were diagnosed with HP in various forms including chronic HP/IPF, CHP, acute HP, and probable HP. A Canadian study reporting MDT diagnoses found IPF in 27% of patients, unclassified ILDs in 27% of patients, HP in 21% patients, and other smoking-related ILDs in 10% of patients. 13 20 The difference from our observations may reflect variation in the risk factors in the populations studied. Unclassified ILDs, including ILA, were reported in 8.4% of patients, which is comparable to previously published data. 20

HP, either as a definite diagnosis or a probable diagnosis overlapping with other ILDs, was diagnosed in approximately 35% of our total cohort. This is higher than in the study reported by Walsh. 21 HP is a great mimic and the consensus diagnoses made following an MDT discussion reflect the challenges that clinicians face when managing patients with ILD in routine practice. Indeed, previous studies have shown that the diagnosis of IPF can be wrong in around 50% of cases after a thorough search for risk factors, exposures, and related fibrosis. 19 22 23

A diagnosis of HP was made based on environmental exposure history, serological testing, bronchoscopy with bronchoalveolar lavage (BAL), and surgical lung biopsy data. It has been suggested that the number of lung biopsies performed increases with more regular MDT meetings. 24 This may reflect the desire of ILD-MDT members for more certainty in diagnosis and management.

The spectrum of diagnoses in our cohort is similar to that reported by Richeldi et al. 25 They reported that academic centers made more accurate ILD diagnoses and performed fewer biopsies than nonacademic hospitals. 25 A recent study also suggested that ILD-MDT meetings remove the need for a lung biopsy in many patients with DPLD. 26

The management of DPLD is strongly influenced by the early provisional diagnosis made by the ILD-MDT. This provisional diagnosis also frames the team's recommendations for the initial choice of pharmacological and nonpharmacological therapies. The early involvement of a rheumatologist in the management of ILD was shown to be useful by Walsh et al. 27 Thus, a consensus agreement between rheumatologists and pulmonologists is needed for the diagnosis of CTD ILDs and the initiation of systemic steroids alongside other immunosuppressants and/or steroid sparing agents in this subgroup. In the case of IPF, the choice of antifibrotic therapy is recommended by the members of the ILD-MDT meetings. This approach has been used in other studies showing the benefit of ILD-MDTs in the management of patients with CTD-ILDs. 28 29

In our cohort, when the diagnosis was unclear and the ILD-MDT could not make a definitive diagnosis, the choice of therapy was based on the most likely working diagnosis achieved by consensus. These patients would receive regular follow-up with a pulmonologist and a rheumatologist to monitor the response to therapy. All other patients would only be followed by a pulmonologist.

In this cohort, 35% received steroid and MMF, 18% received Pred-azathio, 18% received antifibrotic therapy alone, and 27% required oxygen therapy. The behavior of the disease after the ILD-MDT meeting was an important factor in defining treatment response and the need to adjust medications at subsequent follow-up. For example, lack of response to steroids and immunosuppressants could result in a change of therapy to an antifibrotic or combination therapy. Other management decisions including the need for oxygen therapy, pulmonary rehabilitation, and referral for lung transplantation were also taken at follow-up clinic visits. The use of pulmonary rehabilitation, exercise, and oxygen therapy are encouraged by ILD-MDT meetings. 30 31

The main limitations of our study are its retrospective nature and the collection of data from a single center. Furthermore, the patients were only presented to the ILD-MDT meetings once and longitudinal follow-up data were not available to determine the patients' course thereafter. This would have informed us on the behavior of the working diagnosis and subsequent change in the management decisions in the cases where initial diagnosis was changed.

## Conclusion

Discussion of complex cases in ILD-MDT can increase the accuracy of diagnoses and treatment certainty. 17 25 27 As the management of ILD remains extremely challenging, more data are needed to guide clinicians and achieve the maximum benefit from ILD-MDT meetings for patient outcomes. Therefore, in the present study, we describe the diagnoses and management decisions made at the ILD-MDT meetings at our institution.
